# Dexmedetomidine administration in a patient with status epilepticus under color density spectral array monitoring

**DOI:** 10.1186/s40981-019-0234-1

**Published:** 2019-02-27

**Authors:** Shinju Obara, Koh Kakinouchi, Jun Honda, Yoshie Noji, Chie Hanayama, Masahiro Murakawa

**Affiliations:** 1 Surgical Operation Department, Fukushima Medical University Hospital, 1 Hikarigaoka, Fukushima, 960-1295 Japan; 2 Department of Anesthesiology, Fukushima Medical University Hospital, 1 Hikarigaoka, Fukushima, 960-1295 Japan; 3 Department of Anesthesiology, Fukushima Medical University School of Medicine, 1 Hikarigaoka, Fukushima, 960-1295 Japan

**Keywords:** Dexmedetomidine, Status epilepticus, Anesthesia, Color density spectral array, Processed electroencephalogram

## Abstract

**Background:**

Status epilepticus requires immediate treatment because treatment delay can cause permanent neurologic complications. Dexmedetomidine may be an option for the treatment of status epilepticus although its effect remains unclear with conflicting reports.

**Case presentation:**

A 64-year-old woman with epilepsy with complex partial seizures underwent total knee arthroplasty. After emergence from general anesthesia, she developed status epilepticus and was transferred to the intensive care unit. Following initial treatment using benzodiazepines, phenytoin, and levetiracetam, dexmedetomidine (0.37 μg/kg loading in 10 min followed by 0.6 μg/kg/h) was administered and seizures terminated in 20 min. Color density spectral array using Root® with SedLine® (Masimo, Irvine, CA, USA) showed an increase in power in high frequency band of the electroencephalogram during the seizure attacks.

**Conclusion:**

We described a case of status epilepticus which was treated with dexmedetomidine and monitored using color density spectral array.

## Background

Status epilepticus, considered the most extreme form of seizure, requires immediate treatment, usually with benzodiazepines, because treatment delay can cause permanent neurologic complications [1]. Epilepsy patients may suffer seizures with convulsions at emergence from general anesthesia [2], even if the disease is stabilized before the induction of anesthesia [3]. Dexmedetomidine, a highly selective α-2-adrenoceptor agonist, has sedative and analgesic effects without respiratory depression and is used perioperatively. The effect of dexmedetomidine on seizures remains unclear with conflicting reports although it may be an option for the treatment of status epilepticus. Color density spectral array (CDSA) that is displayed on processed electroencephalogram (EEG) monitors shows the power spectrum over time of the EEG, which may be useful to monitor patients with seizures.

Here, we describe a case of status epilepticus after emergence of general anesthesia in which dexmedetomidine was administered and the effect was observed using CDSA.

## Case presentations

**Fig. 1:**
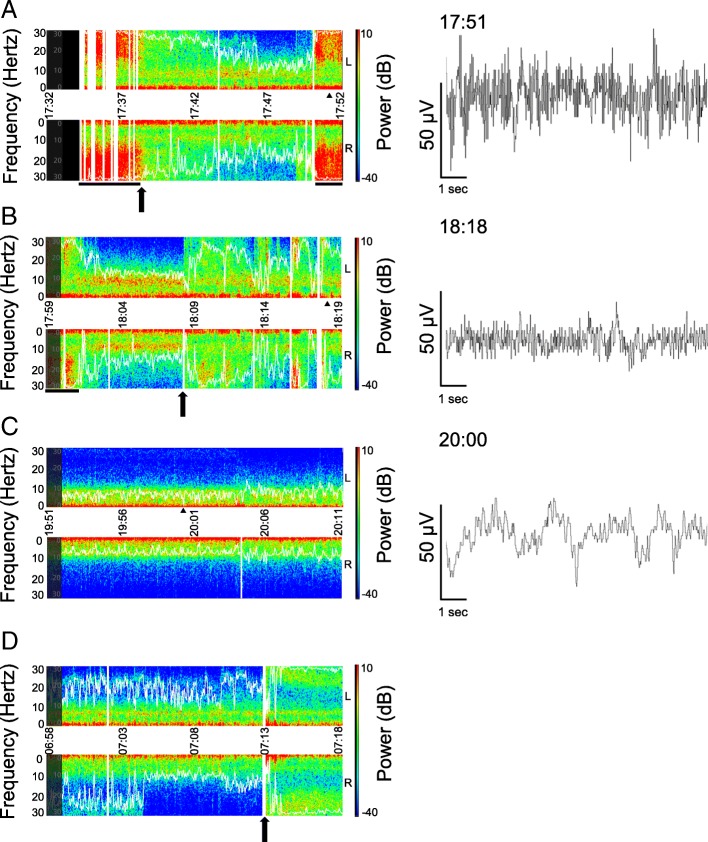
Screen captures of color density spectral array (CDSA) in the intensive care unit. The upper and lower portions of left panels represent EEG power spectrum obtained from the left and right forehead, respectively. *X*-axis represents time. *Y*-axes represent frequency (Hz). The farther from the center horizontal line the higher the frequency. The right vertical line represents power of EEG (dB) where warmer colors represent higher power. White curves represent spectral edge frequency 95%, which is the frequency below which 95% of the spectral power of an EEG resides. Vertical white bars represent missing data on CDSA due to artifacts. Right panels represent 10-s EEG in the left front polar (fp1) region, corresponding to selected time points (black triangle) in left panels. **a** CDSA immediately after the patient entered the ICU. A black arrow represents the time of administration of diazepam 5 mg iv. Black horizontal bars represent convulsions. **b** A black arrow represents the start of dexmedetomidine administration. **c** Approximately 2 h after the start of dexmedetomidine infusion. Patient state index was about 23. **d** CDSA next morning. A black arrow represents the spontaneous awakening. PSI increased from 40 to 83 in a minute. Corresponding EEG of **d** could not be downloaded due to a technical failure. Although **d** shows an asymmetric CDSA, the cause is unknown. Raw EEG waves were illustrated by EDFbrowser 1.64 (https://www.teuniz.net/edfbrowser/; last accessed on February 11, 2019) using “.edf” files downloaded from Root® system

Written informed consent was obtained from the patient for publication of this case report. A 64-year-old woman (body weight, 72 kg; height, 155 cm) with a history of epilepsy with complex partial seizures developed right knee osteoarthritis and was scheduled for total knee arthroplasty. One year previously, she underwent the same operation on the contralateral side and developed tonic-clonic convulsions after emergence of general anesthesia in the operating room and the ward. She was then treated with repeated diazepam administration; however, her postoperative management became difficult due to repeated respiratory arrests. Her epilepsy control improved subsequently although seizure attacks occurred approximately once a week, according to her medical record. She was under medication of valproate sodium, carbamazepine, and levetiracetam, each of which blood concentrations was in a therapeutic range. Preoperative examinations revealed no other abnormal findings except for an increase in γ-glutamyltransferase (61 U/L) probably due to medication. On the morning of surgery, she took usual anticonvulsants and received no anesthetic premedication. Patient monitoring included continuous electrocardiography, pulse oximetry (SpO_2_), capnometry, and non-invasive blood pressure. Patient state index was also monitored using Root® with SedLine® (Masimo, Irvine, CA, USA; version 2000). After femoral nerve block with 20 mL of 0.375% ropivacaine, general anesthesia with tracheal intubation was induced with propofol 80 mg, remifentanil 0.19 μg/kg/min, and rocuronium 50 mg, and maintained with sevoflurane 1.3% and remifentanil 0.14–0.23 μg/kg/min. During the anesthesia, SpO_2_ was maintained at 97% or more and end-tidal CO_2_ was kept between 36 and 41 mmHg (more than 39 mmHg at most time points during the last hour). Patient state index were between 22 and 27. For postoperative analgesia, acetaminophen 1 g and flurbiprofen axetil 50 mg were intravenously administered. The surgery was completed without incident, and after the recovery of consciousness and spontaneous respiration, patient’s trachea was extubated. Tonic muscular contraction was observed in the upper limbs, body trunk, neck, and face along with respiratory depression. SpO_2_ decreased from 100 to 88%. Although these symptoms were temporarily relieved by midazolam 3 mg iv, they recurred in a few minutes. Additional midazolam 6 mg and phenytoin 125 mg were administered in the recovery room, but the symptoms did not improve, and the patient was transferred to the intensive care unit (ICU). Levetiracetam 500 mg was administered by intravenous drip. In addition, a total of 15 mg diazepam was administered intermittently during the first hour and the patient often required respiratory assistance with jaw thrust. However, convulsions persisted. CDSA showed a spread of warmer colors (i.e., higher power) in a wide frequency band of the EEG during the convulsions (Fig. 1a) with PSI > 80. She could not rest in bed and complained of knee pain once with dizziness. Dexmedetomidine (0.37 μg/kg loading in 10 min followed by 0.6 μg/kg/h) was administered, and she was sedated in 20 min. During the improvement from the convulsion, CSDA showed a decrease in warmer colors in a wide frequency band and raw EEG showed decreased amplitude (Fig. 1b). After termination of convulsions, in the CDSA, blue tones, which indicate a drop in EEG power, became predominant (Fig. 1c). Dexmedetomidine administration was continued until the next morning while gradually decreasing the dose to 0.2 μg/kg/h (Fig. 1d). No cardiopulmonary suppression or recurrence of convulsions that required intervention was observed. Blood test revealed no electrolyte disturbance. She was unconscious after induction of general anesthesia until the next morning. She had convulsions once on the eighth day after surgery without major problems and was discharged on the 20th day.

## Discussion

The Japanese guidelines for epilepsy treatment [4] state that a seizure lasting 5 min or longer is considered as status epilepticus and initial treatment should be started. The first-line treatments for status epilepticus include administration of benzodiazepines (stage I treatment), followed by fosphenytoin, phenytoin, and levetiracetam, among others (stage II treatment). In case of insufficient effect, general anesthesia is performed using propofol, barbiturate, or midazolam (stages III and IV treatments). However, benzodiazepines and barbiturates have a respiratory depression effect, and general anesthesia requires tracheal intubation and mechanical ventilation. Therefore, anticonvulsants that do not cause respiratory depression are preferable. Anticonvulsant property of dexmedetomidine has been reported. In animal studies, dexmedetomidine has increased the seizure threshold [5, 6]. Using an in vitro rat model, Kurosawa et al. demonstrated that the anticonvulsant effect of dexmedetomidine is mediated mainly via α-2-adrenoceptors and imidazoline type 1 and 2 receptors are involved in the effect of dexmedetomidine on the epileptiform activity [7].

On the other hand, contradictory results about the effects of dexmedetomidine on seizures have been reported. Talke et al. revealed that dexmedetomidine did not have a statistically significant effect on interictal epileptiform activity in patients with refractory seizure disorders [8]. Oda et al. showed that dexmedetomidine did not affect spike activity in patients with temporal lobe epilepsy anesthetized with sevoflurane [9]. Furthermore, a decrease in the seizure threshold by dexmedetomidine in animal studies has been reported [10, 11]. In neonates, dexmedetomidine-induced epileptic seizures were reported [12].

The inhibition of central noradrenergic transmission facilitates seizure expression [10]. Dexmedetomidine agonistically acts on presynaptic and postsynaptic α-2-adrenoceptors. If dexmedetomidine dominantly acts on the presynaptic α-2- adrenoceptors, proconvulsive actions with a reduction in noradrenaline release due to the negative feedback system can induce seizure. Contrary to the presynaptic effect, preferential binding to postsynaptic α-2- adrenoceptors would enhance noradrenaline-mediated postsynaptic activity [10], and anticonvulsant action could be produced. Thus, effects of dexmedetomidine on epilepsy could be dependent on which synaptic site is dominant. The dominance may be dependent on patients and situations, and a method to clinically control the dominance has not been reported. In the current case, the anticonvulsant action was observed that may be associated with the above-mentioned mechanism, under a clinical dose of dexmedetomidine.

In the current case, the patient developed seizures after emergence from anesthesia. We decided to manage the patient in the ICU as we expected that initial treatment would not produce sufficient effect according to her past history and that general anesthesia would be required. The seizures stopped after administration of benzodiazepine but recurred within 10 min, and this was repeated. Dexmedetomidine was initially used to reduce her knee pain and the risk associated with body motion, which showed anticonvulsant effect, probably due to interaction with other drugs which had already been administered. Although in a rat model, plasma dexmedetomidine concentration levels at which loss of the righting reflex is induced were associated with a decrease in convulsive seizures caused by local anesthetics [5], pharmacodynamics (i.e., relationship between plasma or effect-site concentration vs clinical effect) of the anticonvulsant effect of dexmedetomidine in humans is still unclear. In terms of sedative effect, according to pharmacokinetic and pharmacodynamic simulations using a recently published model [13], simulated bispectral index decreases from 97 to 80 in the first 10 min and then stabilizes around 75 in the following few hours (i.e., equivalent to “light sedation”), which agrees with the current course of treatment. Assuming that anticonvulsive and sedative effects are produced in similar effect-site concentrations of dexmedetomidine even in humans, we can consider that dexmedetomidine worked as an anticonvulsant to some extent in the current case. The patient was to undergo general anesthesia if dexmedetomidine was not effective; however, as a result, she could avoid tracheal intubation. The loading dose of dexmedetomidine was less than the recommended dose (1 μg/kg in 10 min) to avoid bradycardia because the heart rate was around 50/min.

CDSA shows time on the *x*-axis, frequency on the *y*-axis, and the color spectrum ranged from blue (minimum power) to dark red (maximum power) (see figure). Seizure evolution may involve increases in frequency and amplitude in EEG and thus may appear on CDSA images as upward arches on the *y*-axis (increased frequency) in warmer colors (increased power) [14]. In the current case, CDSA showed high power (i.e., high amplitude in raw EEG as shown in the right panel of Fig. 1a) in the high-frequency range during convulsions. Therefore, after the termination of convulsions by administration of dexmedetomidine in the ICU, we planned to increase the dexmedetomidine dose or to use benzodiazepines as a rescue therapy in case CDSA showed such patterns or convulsions occurred; however, this was not required. Thus, the CDSA obtained using a processed EEG monitor may be useful for perioperative monitoring of epileptic patients, especially of those with non-convulsive status epilepticus of which the difficulty in visual diagnosis can cause delay in treatment. However, CDSA changes should be carefully examined because of possibilities of false-positive results caused by various artifacts including electromyograms and body movements. In addition, for the evaluation of epilepsy treatment under general anesthesia, the disappearance of convulsions and epileptiform discharge on EEG are required [4]. Furthermore, the maintenance of flat EEG [15] or burst suppression on EEG [16] may contribute to better outcomes. For these monitoring, CDSA obtained using processed EEG monitors with a limited number of channels is inadequate, and continuous monitoring with conventional EEG is required.

## Conclusion

In this case report, we presented a case of status epilepticus which was treated with dexmedetomidine and monitored using CDSA. This strategy may be an option for the management of patients with status epilepticus.

## Abbreviations

CDSA: Color density spectrum array
EEG: Electroencephalogram
PSI: Patient state index
