# Correction to: Dexmedetomidine administration in a patient with status epilepticus under color density spectral array monitoring

**DOI:** 10.1186/s40981-019-0242-1

**Published:** 2019-03-23

**Authors:** Shinju Obara, Koh Kakinouchi, Jun Honda, Yoshie Noji, Chie Hanayama, Masahiro Murakawa

**Affiliations:** 1 Surgical Operation Department, Fukushima Medical University Hospital, 1 Hikarigaoka, Fukushima, 960-1295 Japan; 2 Department of Anesthesiology, Fukushima Medical University Hospital, 1 Hikarigaoka, Fukushima, 960-1295 Japan; 3 Department of Anesthesiology, Fukushima Medical University School of Medicine, 1 Hikarigaoka, Fukushima, 960-1295 Japan

**Correction to: JA Clin Rep (2019) 5:12**

**https://doi.org/10.1186/s40981-019-0234-1**

Following publication of the original article [1], the authors reported an error in Fig. 1b and c. A black bar and arrows were added.

The publishers apologise for this error. The original article [1] has been updated.

**Fig. 1:**
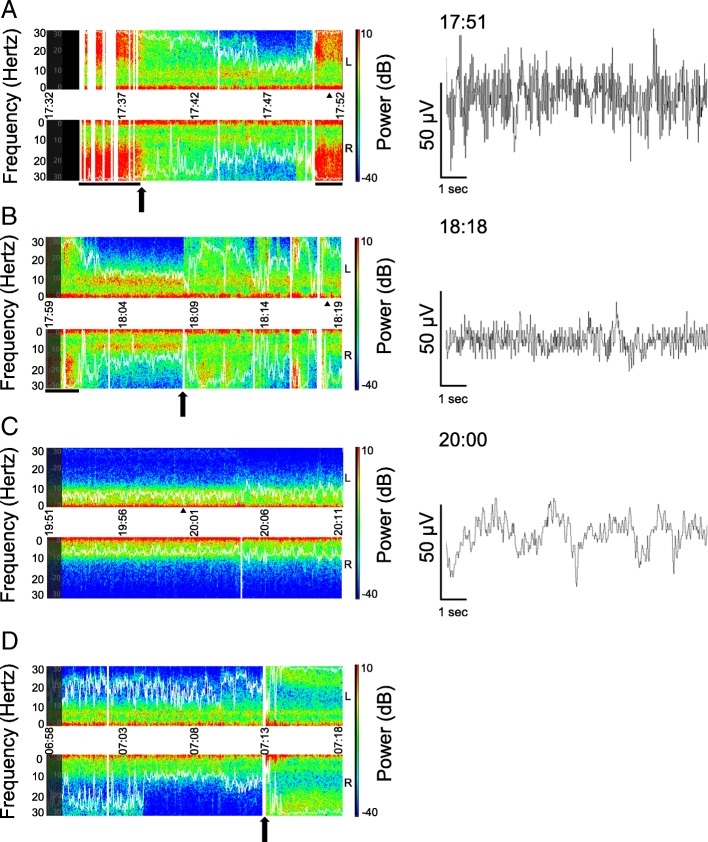
Screen captures of color density spectral array (CDSA) in the intensive care unit. The upper and lower portions of left panels represent EEG power spectrum obtained from the left and right forehead, respectively. X-axis represents time. Y-axes represent frequency (Hz). The farther from the center horizontal line the higher the frequency. The right vertical line represents power of EEG (dB) where warmer colors represent higher power. White curves represent spectral edge frequency 95%, which is the frequency below which 95% of the spectral power of an EEG resides. Vertical white bars represent missing data on CDSA due to artifacts. Right panels represent 10-s EEG in the left front polar (fp1) region, corresponding to selected time points (black triangle) in left panels. **a** CDSA immediately after the patient entered the ICU. A black arrow represents the time of administration of diazepam 5?mg iv. Black horizontal bars represent convulsions. **b** A black arrow represents the start of dexmedetomidine administration. **c** Approximately 2?h after the start of dexmedetomidine infusion. Patient state index was about 23. **d** CDSA next morning. A black arrow represents the spontaneous awakening. PSI increased from 40 to 83 in a minute. Corresponding EEG of d could not be downloaded due to a technical failure. Although d shows an asymmetric CDSA, the cause is unknown. Raw EEG waves were illustrated by EDFbrowser 1.64 (https://www.teuniz.net/edfbrowser/; last accessed on February 11, 2019) using “.edf” files downloaded from Root® system
